# Trends in the epidemiology of depression and comorbidities from 2000 to 2019 in Belgium

**DOI:** 10.1186/s12875-022-01769-w

**Published:** 2022-06-28

**Authors:** Roosje Walrave, Simon Gabriël Beerten, Pavlos Mamouris, Kristien Coteur, Marc Van Nuland, Gijs Van Pottelbergh, Lidia Casas, Bert Vaes

**Affiliations:** 1 Department of Public Health and Primary Care, Kapucijnenvoer 7 blok h, bus 7001 KU Leuven, Leuven, Belgium; 2 Social Epidemiology and Health Policy (SEHPO), Department of Family Medicine and Population Health (FAMPOP), University of Antwerp, Antwerp, Belgium; 3 Institute for Environment and Sustainable Development (IMDO), University of Antwerp, Antwerp, Belgium

**Keywords:** Epidemiology, Depression, Flanders, Belgium

## Abstract

**Background:**

Depression is a common mental disorder in family practice with an impact on global health. The aim of this study is to provide insight in the trends of epidemiological measures as well as pharmacological treatments and comorbidities of depression.

**Methods:**

A study using data from INTEGO, a family practice registration network in Flanders, Belgium. Trends in age-standardized prevalence and incidence of depression from 2000 to 2019 as well as antidepressant prescriptions in prevalent depression cases were analyzed with join point regression. Comorbidity profiles were explored using the Cochran-Armitage test and the Jonckheere-Terpstra test.

**Results:**

We identified 538 299 patients older than 15 years during the study period. We found an increasing trend in the age-standardized prevalence of depression from 6.73 % in 2000 to 9.20 % in 2019. For the incidence of depression, a decreasing trend was observed from 2000 to 2015 with an incidence of 9.42/1000 in 2000 and 6.89/1000 in 2015, followed by an increasing trend from 2015 to 2019 (incidence of 13.64/1000 in 2019). The average number of chronic diseases per patient with depression increased significantly during the study period (from 1.2 to 1.8), and the proportion of patients relative to the whole study population that received at least one antidepressant prescription per year increased between 2000 and 2019 from 26.44% to 40.16%.

**Conclusions:**

The prevalence of depression increases while the incidence sharply rises, but only in recent years. Patients with depression tend to have more comorbidities, making a multi-faceted approach to these patients more important.

**Supplementary Information:**

The online version contains supplementary material available at 10.1186/s12875-022-01769-w.

## Introduction

Depression is a mental disorder with an important impact on global health [1]. It is the third leading cause of non-fatal health loss according to the World Health Organization (WHO). In 2019, globally more than 279 million people were affected by depression [1]. The 2018 Belgian health survey, using self-reported questionnaire data, showed an estimated prevalence of depression of 9.4% for people aged 15 years and older [2]. According to the Global Burden of Disease Study in 2019 (using Diagnostic and Statistical Manual of Mental Disorders and International Classification of Diseases 10 criteria), depression prevalence in Belgium was 4.36%, comparable with the surrounding countries, with France having the highest prevalence (4.74%) [1]. This contrasts with the global prevalence of depression, which was estimated to be 3.76%. With regard to time trends, the majority of studies reported an increase from the early 2000s till recent years [3–9].

However, there are inconsistencies across age groups. Some studies show increasing trends in prevalence in young adults and no change or even a decline in older adults [4, 7, 8], while others also found an increase in the middle-aged or elderly population [6, 9].

The use of mental health services and the prescription of antidepressants seem to have consistently increased in the last two decades [6, 10–16]. The type of antidepressant prescribed changed, with a decline in the use of tricyclic antidepressants (TCAs) and a rise of selective serotonin and noradrenalin reuptake inhibitors (SNRIs) and selective serotonin reuptake inhibitors (SSRIs) [10, 16].

There is also a rising problem of depression associated with multimorbidity [6, 17–19]. Individuals with depression are more likely than individuals without depression to have comorbid physical conditions [17, 20]. Moreover, depression is two to three times more likely in patients with multimorbidity compared to patients without [18].

While many studies have covered these topics, an analysis of data across age categories in family practice, with its unique patient population, is missing in literature. In order to have an accurate idea of how depression is represented in this context, we focused on tangible data, such as diagnoses made by the physicians or prescriptions given out, something which has already been done before in different settings [21, 22].

In this study we provide a comprehensive overview of the trends in the epidemiology of depression in family practice, its drug treatment and comorbidities in patients with depression between 2000 and 2019 in Flanders, Belgium.

## Methods

### Study design and data collection

Data for this study were obtained from INTEGO, a Belgian family practice morbidity registration network managed at the Department of General Practice of the University of Leuven [23]. The registry started in 1994 and was founded to inform public health on the incidence and prevalence of disease in family practice.

In 2019, over 300 family physicians (FPs) evenly spread throughout Flanders, Belgium, were participating in the INTEGO project, which now provides data from over 400 000 patients. Family practices apply for inclusion in the registry. Before acceptance of their data, registration performance is audited using algorithms to compare their results with those of all other applicants. Only data from practices with optimal registration performance are included in the database. Additionally, INTEGO data are externally validated by means of national and international comparisons [23]. INTEGO FPs prospectively and routinely register all new diagnoses and new drug prescriptions using computer-generated keywords internally linked to codes.

New data are encrypted and collected from the FPs’ personal computers and entered in a central database on a weekly basis. We excluded data from 2020 as the currently ongoing COVID pandemic might change epidemiologic trends outside the scope of this paper.

New diagnoses are classified according to the International Classification of Primary Care 2 (ICPC-2) and International Statistical Classification of Diseases and Related Health Problems, 10th Revision. Drugs are classified according to the WHO’s Anatomical Therapeutic Chemical (ATC) classification system. The denominator is the yearly contact group (YCG). These are the patients who visit a certain practice at least once in a given year [23, 24]. They have a unique pseudonymized patient ID (based on their national social security number), which remains the same across practices. Data from family practices outside the INTEGO network are not included in the database.

### Depression, comorbidities and antidepressive treatment

Patients with depression were identified based on the ICPC-2 coded diagnosis P76 “Depression” in their Electronic Medical Record (EMR). Cases were considered prevalent if a P76 diagnosis was ever registered without considering if the patient had an active depressive episode or whether the patient had been free of depressive episodes for years. In other words, we considered depression as a chronic disease. Patients were no longer considered prevalent if they stopped being included in the YCG (i.e. deceased, moved to practice outside of the INTEGO network). No distinction could be made between mild, moderate and severe depression. Cases were considered incident if a first diagnosis was made the same year.

A disease count was calculated for all incident depression cases for which a list of chronic diseases was used (Table A1, Appendix). For the presence of chronic kidney disease, the glomerular filtration rate was estimated based on the closest creatinine measurement in the 2 years before or after the date of diagnosis of depression.

Medication for patients with depression was recorded for all prevalent cases each year between 2000 and 2019. Medication use in a specific year was considered positive when at least one prescription had been made in that year (Table A2, Appendix).

### Data analysis

Prevalence (/100 patients) and incidence (/1000 patients) were calculated for patients with depression by gender.

The rates were age-standardized by taking the Flemish population in Belgium as the standard population (reference year 2000). Age groups were formed starting from 15-29 years, 30-44 years, 45-59 years, 60-74 years, with 75 years and older being the last group for standardization. Additionally, the trend in age-standardized rates between 2000 and 2019 was analyzed. For that purpose, a join point regression analysis was performed [25]. From the join point regression model, the annual percentage change (APC) and the average annual percentage change (AAPC) were extracted. The APC is calculated for each significant trend from a piecewise log-linear model on the logarithm of the age-standardized rate versus the year. The AAPC represents the average of APC estimates per significant trend weighted by the corresponding trend length (number of years in the trend). The points between each trend period are called join points, which represent a significant change in the calculated trend (either upwards or downwards) and can be different across strata. This implies that the number and length of trend periods can vary between strata as well.

The trend analysis using the join point regression model was performed using the SEER*Stat software [Join Point Trend Analysis software from the Surveillance Research Program of the US National Cancer Institute (available at http://surveillance.cancer.gov/joinpoint)]. Trends in comorbidity profiles were explored in incident depression cases with the Cochran-Armitage test and the Jonckheere-Terpstra test over the following intervals: 2000–2003, 2004-2007, 2008-2011, 2012-2016 and 2017-2019. Trends in prescription of antidepressants over the years 2000–2019 were analyzed using a join point regression analysis, as described above. A two-sided *P* value <0.05 was considered to be statistically significant. These analyses were performed using R Software V.4.8.0.1 (Free Software Foundation, Boston, Massachusetts, USA) (*DescTools* and *clinfun* packages).

## Results

### Trends in age-standardized prevalence and incidence of depression (2000-2019)

There were 538 299 unique patients older than 15 during the study period. The age-standardized prevalence of depression increased over time.

**Fig. 1:**
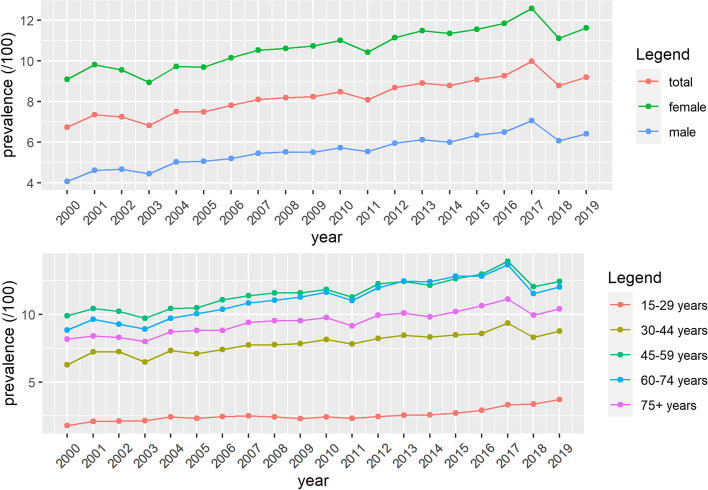
Prevalence of depression in Flanders, Belgium from 2000 to 2019, age-standardized (**A**) and per age group (**B**)

A different trend in women and men was noted. Although women had a higher prevalence of depression during the whole study period, the AAPC for men was higher (Table 1, Fig. 1). The prevalence of depression differed in different age groups. The highest prevalence was found in patients aged 45-59. A significant rise in prevalence was observed in all age groups. The highest AAPC was found in the youngest group, with the steepest rise between 2013-2019 (Table 1, Fig. 1).

**Table 1:** Trends in the prevalence and incidence of depression in Flanders, Belgium (2000-2019)

	Summary			Trend 1		Trend 2		Trend 3	
	Year 2000	Year 2019	AAPC (95% CI)	Years	APC (95% CI)	Years	APC (95% CI)	Years	APC (95% CI)
Prevalence (/100)									
Total	6.73	9.20	1.6 (1.2;1.9)	2000-2019	1.6 (1.2;1.9)				
Women	9.10	11.63	1.3 (1.0;1.6)	2000-2019	1.3 (1.0;1.6)				
Men	4.05	6.40	2.0 (1.6;2.5)	2000-2019	2.0 (1.6;2.5)				
15-29	1.80	3.72	3.6 (2.7;4.6)	2000-2004	6.4 (2.4;10.7)	2004-2013	0.2 (-0.9;1.3)	2013-2019	7.2 (5.7;8.6)
30-44	6.30	8.77	1.4 (1.1;1.8)	2000-2019	1.4 (1.1;1.8)				
45-59	9.90	12.44	1.1 (0.5;1.7)	2000-2017	1.7 (1.4;2.0)	2017-2019	-3.8 (-9.3;2.1)		
60-74	8.86	12.04	1.5 (0.6;2.3)	2000-2016	2.4 (1.9;3.0)	2016-2019	-3.6 (-8.3;1.4)		
75+	8.17	10.40	1.3 (1.0:1.6)	2000-2019	1.3 (1.0:1.6)				
Incidence (/1000)									
Total	9.42	13.64	1.9 (0.4;3.3)	2000-2015	-2.3 (-3.3;-1.3)	2015-2019	19.1 (11.8;26.9)		
Women	11.62	16.68	1.7 (0.0;3.5)	2000-2015	-2.5 (-3.7;-1.2)	2015-2019	19.2 (10.3;28.7)		
Men	7.14	10.27	1.6 (0.4;2.9)	2000-2014	-2.5 (-3.5;-1.5)	2014-2019	14.2 (9.6;19.0)		
15-29	7.37	13.62	3.3 (1.1;5.5)	2000-2015	-1.0 (-2.5;0.6)	2015-2019	20.9 (10.4;32.4)		
30-44	10.66	16.66	1.7 (0.0;3.5)	2000-2015	-1.8 (-3.0;-0.6)	2015-2019	16.2 (7.8;25.3)		
45-59	11.94	14.89	1.4 (-0.5;3.3)	2000-2015	-2.7 (-3.9;-1.4)	2015-2019	18.1 (8.9;28.1)		
60-74	7.67	9.29	0.8 (-1.7;3.2)	2000-2015	-4.5 (-6.0;-2.9)	2015-2019	23.0 (10.2;37.2)		
75+	7.72	9.80	1.9 (-1.1;4.9)	2000-2014	-3.7 (-6.3;-1.1)	2014-2019	19.5 (8.6;31.6)		

*Abbreviations*: *AAPC* average annual percent change, *APC* annual percent change

**Fig. 2.**
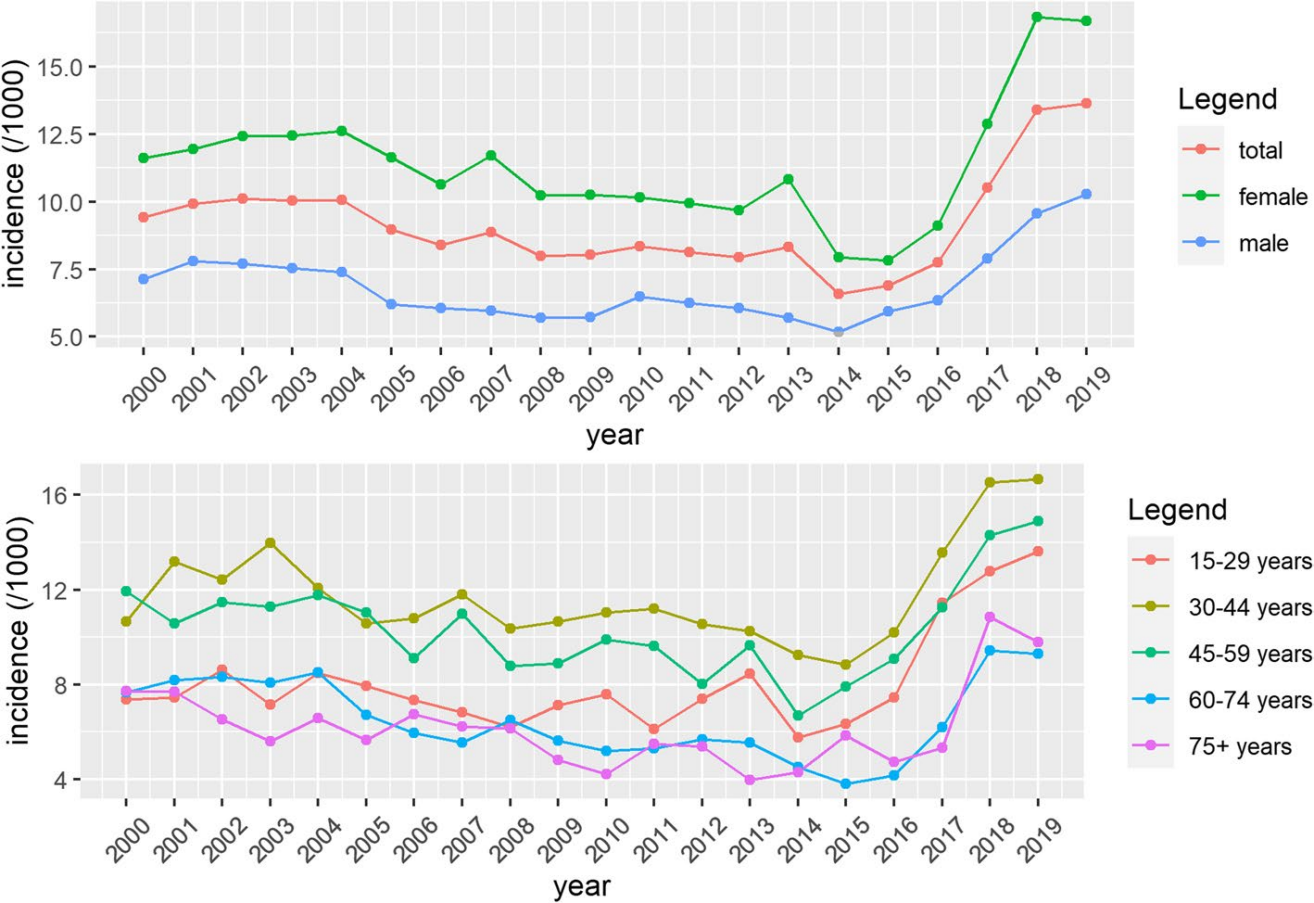
Incidence of depression in Flanders, Belgium from 2000 to 2019, age-standardized (**A**) and per age group (**B**)

The total incidence of depression decreased from 2000 to 2015, after which a steep increase was noted. The incidence also increased steeply and significantly for all age groups, mostly so in age groups 15-29 and 60-74. (Table 1, Fig. 2) The mean age at depression diagnosis did not change significantly during the study period.

### Trends in comorbidities at diagnosis (2000-2019)

The average number of chronic diseases per patient with depression increased significantly over the study period. (Table 2) The leading psychiatric comorbidity was alcohol abuse. In the somatic comorbidities, the strongest rising trend was observed for diabetes mellitus, hypothyroidism, asthma and malignant diseases. The three leading somatic comorbidities for patients with depression in 2017 to 2019 were hypertension, malignancy and asthma, all of which increased significantly during the study period  (Table 2).

**Table 2:** Trends in comorbidities in patients with depression in Flanders, Belgium (2000-2019)

Variables		2000-2003	2004-2007	2008-2011	2012-2016	2017-2019	Trend test	
**STUDY POPULATION**								
Patients with depression (n)		3268	3650	3535	3545	5732	0	
Mean age (standard deviation)		45.2(17)	45.4(17.1)	44.9(17)	44.3(17.5)	44.5(18.6)	1	
Number of women (%)		2054(62.9)	2392(65.5)	2244(63.5)	2189(61.7)	3720(64.9)	0.6175	
**PREVALENCE OF COMORBIDITIES, n (%)**	**ICPC code**							
**Mean chronic disease count per patient (SD)**		1.2(1.6)	1.3(1.8)	1.5(2)	1.6(2)	1.8(2.1)	0.0002	
**Anxiety disorder**	P74	15(0.5)	26(0.7)	28(0.8)	31(0.9)	167(2.9)	<0.0001	
**Alcohol abuse**	P15-16	60(1.8)	98(2.7)	98(2.8)	163(4.6)	208(3.6)	<0.0001	
**Dementia**	P70	15(0.5)	15(0.4)	30(0.8)	41(1.2)	63(1.1)	<0.0001	
Schizophrenia	P72	13(0.4)	15(0.4)	13(0.4)	16(0.5)	28(0.5)	0.4464	
**Suicide/suicide attempt**	P77	3(0.1)	6(0.2)	7(0.2)	9(0.3)	34(0.6)	<0.0001	
**Phobia/compulsive disorder**	P79	29(0.9)	25(0.7)	23(0.7)	46(1.3)	76(1.3)	0.0012	
**Personality disorder**	P80	10(0.3)	26(0.7)	30(0.8)	52(1.5)	194(3.4)	<0.0001	
Anorexia nervosa/bulimia	P86	5(0.2)	1(0)	3(0.1)	5(0.1)	12(0.2)	0	
**Substance abuse**	P18-19	7(0.2)	10(0.3)	17(0.5)	16(0.5)	65(1.1)	<0.0001	
**Atrial fibrillation/flutter**	K78	47(1.4)	50(1.4)	54(1.5)	69(1.9)	128(2.2)	0.0004	
**Hypertension**	K86-87	392(12)	486(13.3)	492(13.9)	429(12.1)	836(14.6)	0.0074	
Heart failure	K77	23(0.7)	35(1)	30(0.8)	32(0.9)	46(0.8)	0.8971	
Atherosclerosis	K92	59(1.8)	82(2.2)	62(1.8)	61(1.7)	88(1.5)	0.0707	
**Ischemic heart disease**	K74-75-76	95(2.9)	126(3.5)	98(2.8)	103(2.9)	140(2.4)	0.039	
**Diabetes Mellitus**	T89-90	114(3.5)	167(4.6)	231(6.5)	320(9)	382(6.7)	<0.0001	
**Hypothyroidism**	T86	50(1.5)	47(1.3)	49(1.4)	57(1.6)	162(2.8)	<0.0001	
**Hyperthyroidism**	T85	6(0.2)	12(0.3)	6(0.2)	7(0.2)	31(0.5)	0.0093	
Irritable bowel syndrome	D01-D93	296(9.1)	363(9.9)	378(10.7)	363(10.2)	549(9.6)	0.6288	
**Asthma**	R96	194(5.9)	277(7.6)	321(9.1)	348(9.8)	603(10.5)	<0.0001	
**COPD**	R95	83(2.5)	110(3)	104(2.9)	94(2.7)	214(3.7)	0.0055	
Osteoarthritis	L89-90-91	314(9.6)	384(10.5)	434(12.3)	356(10)	528(9.2)	0.1349	
**Cerebrovascular disease**	K90-91	60(1.8)	68(1.9)	72(2)	78(2.2)	153(2.7)	0.0025	
**Malignancy**	A79-Y78-N74-Y77-U76-T71-D76-D74-U77-U75-D75-R84-B74-S77-R85-D77-X76-W72-X75-X77	97(3)	139(3.8)	194(5.5)	259(7.3)	696(12.1)	<0.0001	
Chronic Kidney Disease		44(1.3)	35(1)	36(1)	36(1)	66(1.2)	0.7067	

### Trends in antidepressant prescriptions (2000-2019)

**Fig. 3:**
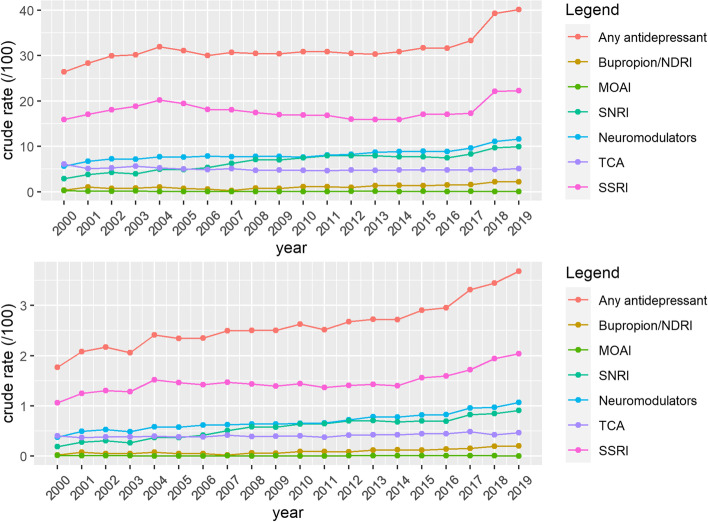
Trends in prescriptions of antidepressants in Flanders, Belgium from 2000 to 2019, in patients with depression (**A**) and relative to the total yearly patient population (**B**). Abbreviations: NDRI = norepinephrine–dopamine reuptake inhibitor, MAOI = monoamine oxidase inhibitors, SNRI = serotonin–norepinephrine reuptake inhibitors, TCA = tricyclic antidepressants, SSRI = selective serotonin reuptake inhibitors

Among prevalent cases with depression, the proportion of patients that received drug treatment increased with one third from 2000 to 2019 (Table 3, Fig. 3). Likewise, the proportion of treated patients who received more than one prescription of an antidepressant per year increased from 63.4% in 2000 to 92.1% in 2019. For the whole population of prevalent cases, the proportion of patients with more than one prescription per year increased from 18% in 2000 to 37% in 2019 (Table 4).

**Table 3:** Trends in first-line treatment in patients with depression in Flanders, Belgium (2000-2019)

	SUMMARY			Trend 1		Trend 2		Trend 3	
Medication (%)	Year 2000	Year 2019	AAPC (95% CI)	Years	APC (95% CI)	Years	APC (95% CI)	Years	APC (95% CI)
Total	26.42	40.16	2.4 (1.0;3.7)	2000-2002	7.1 (-5.4;21.3)	2002-2016	0.2 (-0.3;0.7)	2016-2019	9.6 (5.7;13.6)
Women	26.75	41.92	2.4(1.3;3.6)	2000-2002	8.3 (-2.6;20.5)	2002-2016	0.4 (-0.1;0.8)	2016-2019	8.6 (5.2;12)
Men	25.58	36.51	1.8 (0.9;2.7)	2000-2016	0.1 (-0.5;0.6)	2016-2019	11.6 (6.0;17.5)		
**ANTIDEPRESSANTS**									
SSRI	15.89	22.28	1.8 (0.4;3.1)	2000-2004	5.0 (-0.5;10.8)	2004-2014	-2.4 (-3.6;-1.2)	2014-2019	8.0 (5.3;10.7)
SNRI	2.87	9.95	6.1 (4.6;7.6)	2000-2010	9.1 (7.4;10.8)	2010-2016	-0.4 (-3.4;2.7)	2016-2019	9.7 (3.8; 16.1)
TCA	6.09	5.07	-0.7 (-1.2;-0.1)	2000-2010	-2.0 (-2.8;-1.1)	2010-2019	0.7 (0.0;1.5)		
Neuromodulators	5.62	11.65	3.9 (2.2;5.5)	2000-2002	11.9 (-3.7;30.2)	2002-2016	1.5 (1.0;2.1)	2016-2019	10.0 (5.8;14.3)
MAOI	0.29	0.06	-6.1 (-10.1;-1.9)	2000-2005	-22.8 (-33.6;-10.1)	2005-2019	0,7 (-2.8;4.3)		
Bupropion	0.36	2.19	7.4(5.4;9.5)	2000-2019	7.4(5.4;9.5)				
**OTHER**									
Antipsychotics	7.28	8.29	0.3 (-0.2;0.8)	2000-2019	0.3 (-0.2;0.8)				
Anxiolytics	19.52	18.09	-0.7 (-1.1;-0.2)	2000-2013	-1.2(-1.7;-0.8)	2013-2019	0.5 (-0.7;-1.6)		
Hypnotics and sedatives	11.27	14.77	1.3 (0.8;1.7)	2000-2009	2.0 (1.5;2.5)	2009-2017	-0.1 (-0.7;0.5)	2017-2019	3.5 (0.3;6.8)

**Table 4:** Evolution of antidepressant prescriptions in Flanders, Belgium (2000-2019), using the whole yearly study population as the denominator

Year	One antidepressant prescription (%)	Two or more prescriptions (%)
2000	8.68	17.77
2001	8.39	19.97
2002	8.62	21.31
2003	8.15	22.04
2004	7.68	24.29
2005	7.34	23.75
2006	6.6	23.46
2007	6.77	23.95
2008	6.28	24.23
2009	6.44	23.99
2010	6.94	23.92
2011	6.59	24.31
2012	6.31	24.16
2013	6.28	24.06
2014	6.78	24.07
2015	6.5	25.21
2016	6.74	24.87
2017	6.39	20.48
2018	2.4	36.91
2019	2.83	37.33

In women as well as in men, a significant increase in prescriptions was observed, with a steep rise from 2016 to 2019  (Table 3; Figure A1, Appendix). Regarding the different subclasses of antidepressants, we observed an increase in prescriptions of SNRIs, neuromodulators and bupropion. During the whole study period, SSRIs were the most prescribed antidepressants. For example, in 2019, 22.28% of patients with depression had a prescription for an SSRI. (Table 3) Over the whole study period, prescription rates of antipsychotics remained stable. Prescriptions for anxiolytics showed a slight significant decrease, while those for hypnotics and sedatives showed a small significant increase. Data on those psychopharmaceuticals can be found in Table 3 and in Figure A2, Appendix.

## Discussion

We found an increasing trend in age-standardized prevalence and a first decreasing, then increasing trend in the incidence of depression from 2000 to 2019 in Flanders, Belgium. Among patients diagnosed with depression, the average disease count went from 1.2 to 1.8 comorbidities per patient. The prescription of antidepressive medication among depression-diagnosed patients almost doubled over the study period. SSRIs were prescribed the most, and while there was a significant decrease from 2004 to 2014, afterwards their use increased significantly.

### Trends in the prevalence and incidence of depression

This study showed an increase in age-adjusted prevalence of depression and a decreasing incidence from 2000 to 2015. For the calculation of the prevalence, we have assumed depression to be a chronic condition. While not strictly defined as such, some authors do consider it so [26, 27]. Based on earlier observations of symptom chronicity in depression [28], and the fact that relapse and recurrence are common [29, 30], we have adopted this viewpoint as well.

The trend in prevalence found in this study is consistent with the bulk of existing literature as recently evaluated in a meta-analysis by Moreno-Agostino et al. [31] However, there are not many studies discussing trends in the incidence of depression. Rait et al. found a decrease in the incidence of depression diagnoses in UK primary care from 1996 to 2006 and an increase in depressive *symptoms* [21], something this study did not research. This phenomenon could be explained by medical professionals being more careful with the medicalization of grief and non-pathological feelings of sadness [21, 32]. However, this does not explain the increase in incidence after 2015 that we found.

In light of interpreting our results, it is important to note that the INTEGO database underwent a change in 2017, with the participating practices switching medical software, updating the medical files from one system to another. Conceivably, this might have facilitated coding practice in general, stimulating physicians to code more frequently and diligently. Bearing this in mind, the increase in incidence could also be at least partly explained by a *registration effect*. In essence, this is a sort of registration bias, in that the diagnosis of depression is more likely to be registered than for example 15 years ago. This bias is present in other registries as well [33].

As shown in Fig. 3, the proportional use of SSRIs increased even when using the total yearly study population as a denominator, implying the increase in incidence is at least partially the result of actual morbidity.

Liu et al. also found some similarities with our study [34]. They reviewed global trends in the incidence of depression and found an estimated annual percentage change in Belgium of 0.88 (95% CI = 0.78 to 0.97) from 1990 to 2017, whereas we found an annual percentage change of 1.9 (95% CI = 0.40 to 3.30) from 2000 to 2019. In contrast with our study, they used a linear regression model. Also, their findings were based solely on surveys and self-reported data instead of diagnoses reported by FPs, which might overestimate the burden of depression as compared to actual clinical diagnoses.

We have found several Belgian studies describing the epidemiology of depression, but few focused on trends [35–37]. Wauterickx et al. found an upward trend from 1991 to 1999 based on yearly surveys [38]. On the other hand, the Belgian health interview survey showed a stable prevalence of depression between 2001 and 2018, although a peak of 14.8% was seen in 2013 [2].

In every age group a significant increase in prevalence was noted, with the highest AAPC in the 15 to 29-year-olds. This rise in prevalence in the youngest group has been described in other studies as well [4, 5, 7, 8]. It was also the only age group where no significant decline in the incidence rate was observed. Other studies have suggested that social media and problematic mobile phone use could play a role in this increase of depression [7, 39, 40]. Another explanation could be that this generation has been seeking more help in recent years. Since 2015, for example, there is a national *Red Nose Day* initiative in Belgium [41], focusing on increasing mental health awareness in adolescents. It is important to note in this context that younger patients tend to have worse mental health outcomes than older patients, particularly if they are not in active education or employment [42, 43].

### Trends in comorbidities

Our study noted a rising trend in comorbidities from 2000 to 2019 with a disease count of 1.8 in 2019. An increase of patients with cancer was observed in the population of depressed patients. It is important for FPs and specialists to differentiate depression and non-pathological grief in these patients [44]. To the best of our knowledge, only one study examined time trends of comorbidities in relation to depression. This study, however, differed methodologically from ours in that the presence of comorbidities was linked to self-reported depression severity [45].

In addition, we also noted a rising proportion of depressed patients with alcohol abuse, as well as cardiovascular and metabolic disease. Judging from these results, we could conclude that the depressed patients in our sample became more ‘complex’, as they tended to have more diagnosed comorbidities on average later in the study. Alcohol abuse, for example, tends to be associated with higher drop-out rates from treatment [46]. Taken together, this is likely to impact the treatment of depression in a primary care context, as it will require a more multidisciplinary perspective and approach. On the other hand, part of this increase in comorbidity could be explained by increased detection rather than actual comorbidity. We have mentioned this earlier when discussing the registration effect.

### Trends in medication

Consistently with previous studies [6, 10–16], we observed an increase in the prescription of antidepressive medication among patients diagnosed with depression. Earlier studies in the UK concluded that this might partly be explained by increased chronic prescription [47], something which we did not specifically study.

The decline in prescription of TCAs has also been reported in other studies. TCAs are known to have more side effects than SSRIs and SNRIs [10, 16].

The prescription of SSRIs increased from 2000 to 2004. This can be expected given that they are first choice products because of their safety profile and efficacy [48]. The peak noted in 2004 could be explained by the introduction of escitalopram on the market and the start of reimbursement in Belgium in 2003. From 2004 to 2014 we observed a decline in the prescription of SSRIs, followed by a steep increase from 2014 to 2019. The study of Noordam et al. described a decline in incident SSRI prescriptions, while noting a rise in the prevalence of all antidepressants combined. They attributed this to a shift in the guidelines recommending psychotherapy, especially for milder cases of depression, instead of medication [15].

Other studies noted that patents, marketing and reimbursement of medication could have had a large impact on prescriptions as well. For instance, after 2004 most SSRIs lost their patent and thus marketing for these products declined [12, 49]. On the other hand, reimbursement for SNRIs, bupropion and neuromodulators such as trazodone and mirtazapine, was approved between 2003 and 2008. It is fair to assume that these products were heavily promoted during the study period resulting in the rise of their prescriptions observed in this study.

This is something family physicians should be vigilant about, given that a recent systematic review of scientific evidence did not find the newer medications safer or more efficacious than SSRIs or TCAs [50]. According to the NICE guidelines, SSRIs and TCAs in certain indications are still the first-line treatment, combined with psychotherapy, for moderate to severe depression [51]. For less severe depression, medication should not be used, but psychotherapy should be offered. This approach is also supported by a recent meta-analysis [52].

### Strengths and limitations

The major strengths of this study are the inclusion of a large sample of family practice patients, representative of the general Flemish population [23]. We had two decades of medical information available and because of the very nature of the data collection, information on comorbidities and prescriptions as well. To our knowledge, this is the first comprehensive registry-based study to describe trends in the prevalence and incidence of depression and the first to describe trends in comorbidities and antidepressive medication from 2000 to 2019.

Our study has a few limitations as well. In Belgium, patients do not need to be registered with a particular FP. This means that they have free choice in which FP to consult for new episodes or follow-up. Therefore, our patient population can vary even when the registering FPs stayed the same. In 2016, the Usual Provider Continuity Index was higher than 75% for over 65% of the Belgian population [53]. This means that at least 65% of patients have three out of four family practice visits with their usual FP, instead of with another FP. However, only 40% of patients have an exclusive FP relationship [53], meaning that they only go to their own FP.

The current study used the denominator YCG. Former research has shown that the YCG accounts for 80% of the total practice population [24]. The YCG is not the perfect denominator as it can contain different biases. However, the YCG is the most realistic approach in countries without capitation [24]. Furthermore, by using data from the INTEGO registry we can only extract data registered by FPs in the EMR, with respect to both coded diagnoses and medication prescriptions.

We also do not know which diagnostic tools the FPs used to arrive at the diagnosis and whether they over- or underdiagnosed depression. It was not possible to study an important pillar of depression treatment, namely psychotherapy, since it was not registered in the EMR.

## Conclusion

In this registry-based study of the Flemish population, we noted an increasing trend in the age-adjusted prevalence of depression and a decreasing trend in incidence from 2000 to 2015, followed by a steep increase from 2015 to 2019. A significant rise in the average number of comorbidities at diagnosis was seen. This increased complexity of patients makes the approach to depression in a primary care context more challenging, implying the need for a multidisciplinary approach.

Additionally, there was a rise in the prescription of antidepressants with a steeper increase in recent years, which suggests that first-line treatment of depression in Flanders is still very much dependent on medication.

## Supplementary Information

**Additional file 1.**

## Data Availability

The datasets generated and analysed during the current study are not publicly available due to inclusion of protected health information but can be made available subsequent to further de-identification upon reasonable request to the corresponding author (SGB).

## Abbreviations

AAPC: Average annual percentage change
APC: Annual percentage change
ATC: Anatomical Therapeutic Chemical classification
EMR: Electronic medical record
FP: Family physician
ICPC-2: International Classification of Primary Care 2
MAOI: Monoamine oxidase inhibitor
NDRI: Norepinephrine–dopamine reuptake inhibitor
SNRI: Selective serotonin and noradrenalin reuptake inhibitor
SSRI: Selective serotonin reuptake inhibitor
TCA: Tricyclic antidepressant
WHO: World Health Organization
YCG: Yearly contact group
