# Supplementary material for: Trends in the epidemiology of depression and comorbidities from 2000 to 2019 in Belgium
Source: BMC Prim Care. 2022 Jun 28;23:163. doi: 10.1186/s12875-022-01769-w (PMC9241171; doi:10.1186/s12875-022-01769-w)
Supplement: Supplementary file 1 — Additional file 1. [file 12875_2022_1769_MOESM1_ESM.pdf]

APPENDIX

Table A1: List of codes used in the chronic disease count.

ICPC code	Meaning
A79	Malignancy NOS
A90	Congenital anomaly NOS/multiple
B72	Hodgkin's disease/lymphoma
B73	Leukemia
B74	Malignant neoplasm blood other
B78	Hereditary hemolytic anemia
B83	Purpura/coagulation defect
B90	HIV-infection/AIDS
D74	Malignant neoplasm stomach
D75	Malignant neoplasm colon/rectum
D76	Malignant neoplasm pancreas
D77	Malignant neoplasm digestive other/NOS
F83	Retinopathy
F84	Macular degeneration
F94	Blindness
H83	Otosclerosis
H84	Presbycusis
H86	Deafness
K74	Ischemic heart disease w. angina
K75	Acute myocardial infarction
K76	Ischemic heart disease w/o angina
K77	Heart failure
K82	Pulmonary heart disease
K86	Hypertension uncomplicated
K87	Hypertension complicated
K90	Stroke/cerebrovascular accident
K91	Cerebrovascular disease
K92	Atherosclerosis/PVD
K93	Pulmonary embolism
K94	Phlebitis/thrombophlebitis
L84	Back syndrome w/o radiating pain
L85	Acquired deformity of spine
L88	Rheumatoid arthritis
L89	Osteoarthrosis of hip
L90	Osteoarthrosis of knee
L91	Osteoarthrosis other
L95	Osteoporosis
L98	Acquired deformity of limb
N70	Poliomyelitis
N74	Malignant neoplasm nervous system
N85	Congenital anomaly neurological
N86	Multiple sclerosis
N87	Parkinsonism
N88	Epilepsy
N89	Migraine
N90	Cluster headache
N92	Trigeminal neuralgia

P15	Chronic alcohol abuse
P28	Limited function/disability (p)
P70	Dementia
P71	Organic psychosis other
P72	Schizophrenia
P73	Affective psychosis
P74	Anxiety disorder/anxiety state
P75	Somatization disorder
P77	Suicide/suicide attempt
P79	Phobia/compulsive disorder
P80	Personality disorder
P85	Mental retardation
P98	Psychosis NOS/other
R79	Chronic bronchitis
R84	Malignant neoplasm bronchus/lung
R85	Malignant neoplasm respiratory, other
R95	Chronic obstructive pulmonary disease
R96	Asthma
S77	Malignant neoplasm of skin
S87	Dermatitis/atopic eczema
S91	Psoriasis
S97	Chronic ulcer skin
T71	Malignant neoplasm thyroid
T80	Congenital anomaly endocrine/metabolic
T85	Hyperthyroidism/thyrotoxicosis
T86	Hypothyroidism/myxedema
T89	Diabetes insulin dependent
T90	Diabetes non-insulin dependent
T92	Gout
T93	Lipid disorder
T99	Endocrine/metabolic/nutritional disorder other
U04	Incontinence urine
U75	Malignant neoplasm of kidney
U76	Malignant neoplasm of bladder
U77	Malignant neoplasm urinary other
U85	Congenital anomaly urinary tract
U88	Glomerulonephritis/nephrosclerosis
W72	Malignant neoplasm related to pregnancy
X75	Malignant neoplasm cervix
X76	Malignant neoplasm breast female
X77	Malignant neoplasm genital other (f)
Y77	Malignant neoplasm prostate
Y78	Malign neoplasm male genital other
Y85	Benign prostatic hypertrophy

Table A2: Overview of ATC codes used for medication.

**Total antidepressants**	N06A
SSRIs	N06AB
SNRI	N06AX16, N06AX23, N06AX21, N06AX17
TCA	N06AA
MOAI	N06AF, N06AG
Bupropion	N06AX12
Neuromodulators	N06AX11, N06AX22, N06AX05, N06AX06, N06AX24, N06AX26, N06AX03
**Antipsychotic drugs**	N05A
**Anxiolytic drugs**	N05B
**Hypnotics**	N05C

Table A3: Absolute value reproductions of Tables 1 and 3.

**Table 1**				
	Year 2000		*Year 2019*	
**Prevalence (/100)**				
	Nominator	Denominator	Nominator	Denominator
Total	5533	82782	19646	210988
Women	3978	44161	13314	113980
Men	1555	38621	6329	96961
15-29	339	18786	1724	46376
30-44	1506	23916	4543	51792
45-59	1813	18313	6022	48410
60-74	1241	14011	4820	40027
75+	634	7756	2537	24383
**Incidence (/1000)**				
Total	725	77249	2567	191342
Women	460	40183	1652	100666
Men	265	37066	914	90632
15-29	136	18447	608	44652
30-44	239	22410	787	47249
45-59	197	16500	631	42388
60-74	98	12770	327	35207
75+	55	7122	214	21846
**Table 3**				
**Medication (%)**	*Year 2000*		*Year 2019*	
Total	1463	5533	7765	19335
Women	1065	3978	5483	13081
Men	398	1555	2282	6251
SSRI	880	5533	4307	19335
SNRI	159	5533	1924	19335
TCA	337	5533	981	19335
Neuromodulators	311	5533	2253	19335
MAOI	16	5533	11	19335
Bupropion	20	5533	424	19335
Antipsychotics	403	5533	1602	19335
Anxiolytics	1081	5533	3497	19335
Hypnotics and sedatives	624	5533	2856	19335

Note 1: Male and female patients might not add up to the total, because some patients had no registered gender.

Note 2: The total prevalent patients in 2019 do not correspond between Tables 1 and 3. This is due to a data matching error in some prescriptions for the last three years between our old and new dataset. We did not consider these patients for the medication calculations.

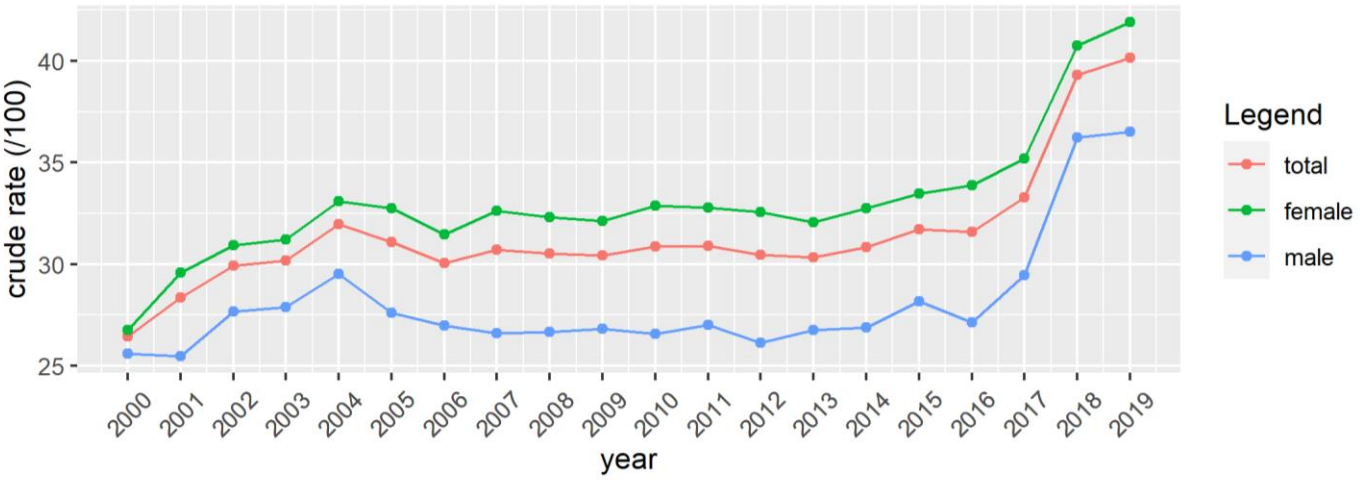

Figure A1. Trends in prescriptions for all antidepressants studied in patients with depression in Flanders, Belgium (2000-2019).

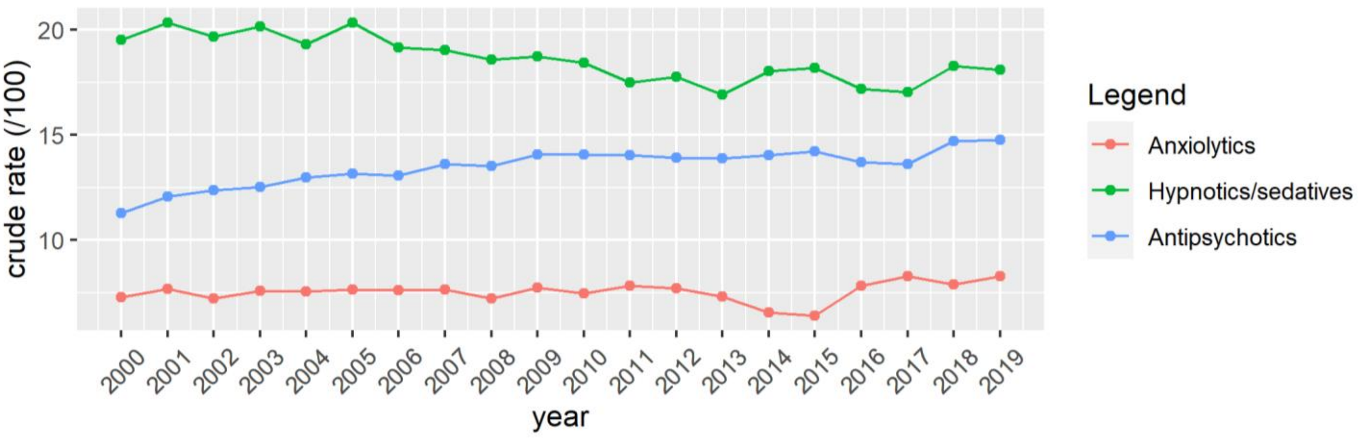

Figure A2. Trends in prescriptions for anxiolytics, hypnotics/sedatives and antipsychotics in patients with depression in Flanders, Belgium (2000-2019).
